# Validity of the Shuttle Walk Test to Assess Cardiorespiratory Fitness in Persons at Varying Stages in the Alzheimer's Continuum

**DOI:** 10.1002/alz70860_102231

**Published:** 2025-12-23

**Authors:** Dereck L. Salisbury, Feng Vankee Lin, Fang Yu

**Affiliations:** ^1^ University of Minnesota, Minneapolis, MN, USA; ^2^ Stanford University, Stanford, CA, USA; ^3^ Arizona State University, Phoenix, AZ, USA

## Abstract

**Background:**

Cardiorespiratory fitness (CRF) has been positively associated with brain volumes and health in older adults and negatively associated with dementia onset or risk and mortality. Cardiopulmonary exercise testing (CPET) is a gold standard test for evaluating CRF for exercise prescription, but requires specialized equipment and is time‐ and resource‐intensive, highlighting the need for more feasible and valid options for evaluating CRF. Therefore, the purpose of this study was to evaluate the validity and relationship of the shuttle walk test (SWT) distance with peak oxygen consumption (VO_2Peak_) from cycle ergometer‐based CPET in persons with amnestic mild cognitive impairment (aMCI) or mild‐to‐moderate AD dementia.

**Method:**

This study used baseline data from two Phase II, single‐blinded clinical trials (The ACT Trial and The FIT‐AD Trial). The sample included 80 participants with aMCI and 90 with mild‐to‐moderate AD. Across the two studies, CRF was measured with VO_2Peak_ obtained from the symptom‐limited peak cycle‐ergometer test and the SWT. Data were analyzed with simple and multiple linear regression. Adjusted models included age, sex, cognition (Montreal Cognitive Assessment [MoCA] or Mini Mental State Examination [MMSE], and body mass index (BMI) that were significantly associated with VO_2peak_.

**Result:**

The participants included 80 from the ACT Trial (55% male, 74.1 [5.7] years, and MoCA 23.2 [2.0]) and 90 from the FIT‐AD Trial (56% males, age 77.1 [6.6] years, and MMSE 21.8 [3.4]). In persons with aMCI, SWT was positively correlated with VO_2Peak_ (*r* = .57 *p* < 0.01). When controlling for age, sex, MoCA, and BMI, SWT distance remained significantly and positively associated with VO_2Peak_ and collectively represented 54% of the variance in VO_2Peak_ (F (5,69) =18.37, *p* <0.001). In persons with AD dementia, SWT was positively correlated with VO_2Peak_ (*r* = .44 *p* <0.01). When controlling for age, sex, MMSE, and BMI, SWT distance remained significantly and positively associated with VO_2Peak_ and collectively represented 43% of the variance in VO_2Peak_ (F (5,77) =11.46, *p* <0.001).

**Conclusion:**

SWT distance is a significant predictor of VO_2Peak_ in persons with cognitive impairment and remains a significant predictor in the presence of related, clinically measured covariates including age, sex, cognition, and BMI.

